# Default-Mode Network Changes in Huntington’s Disease: An Integrated MRI Study of Functional Connectivity and Morphometry

**DOI:** 10.1371/journal.pone.0072159

**Published:** 2013-08-19

**Authors:** Mario Quarantelli, Elena Salvatore, Sara Maria Delle Acque Giorgio, Alessandro Filla, Amedeo Cervo, Cinzia Valeria Russo, Sirio Cocozza, Marco Massarelli, Arturo Brunetti, Giuseppe De Michele

**Affiliations:** 1 Biostructure and Bioimaging Institute, National Research Council, Naples, Italy; 2 Department of Neurosciences, Reproductive Sciences and Odontostomatology, University “Federico II”, Naples, Italy; 3 Department of Advanced Biomedical Sciences, University “Federico II”, Naples, Italy; Hangzhou Normal University, China

## Abstract

Previous MRI studies of functional connectivity in pre-symptomatic mutation carriers of Huntington’s disease (HD) have shown dysfunction of the Default-Mode Network (DMN). No data however are currently available on the DMN alterations in the symptomatic stages of the disease, which are characterized by cortical atrophy involving several DMN nodes. We assessed DMN integrity and its possible correlations with motor and cognitive symptoms in 26 symptomatic HD patients as compared to 22 normal volunteers, by analyzing resting state functional MRI data, using the Precuneal Cortex/Posterior Cingulate Cortices (PC/PCC) as seed, controlling at voxel level for the effect of atrophy by co-varying for gray matter volume. Direct correlation with PC/PCC was decreased, without correlation with atrophy, in the ventral medial prefrontal cortex (including anterior cingulate and subgenual cortex), right dorso-medial prefrontal cortex, and in the right inferior parietal cortex (mainly involving the angular gyrus). Negative correlations with PC/PCC were decreased bilaterally in the inferior parietal cortices, while a cluster in the right middle occipital gyrus presented increased correlation with PC/PCC. DMN changes in the ventral medial prefrontal cortex significantly correlated with the performance at the Stroop test (p = .0002). Widespread DMN changes, not correlating with the atrophy of the involved nodes, are present in symptomatic HD patients, and correlate with cognitive disturbances.

## Introduction

Huntington’s disease (HD) is an autosomal neurodegenerative disorder caused by an expansion of CAG repeats in the gene encoding huntingtin [1]. The clinical phenotype is characterized by motor, cognitive, and psychiatric abnormalities. Main neuropathological changes in HD include prominent loss of medium spiny neurons and atrophy in the striatum, which is a key component of the basal ganglia-thalamocortical circuitry, which serves cognitive and emotional functions [2], [3], besides the motor ones.

Consistent with the known anatomic connections between basal ganglia and the precuneus (which projects to the dorsolateral caudate nucleus and putamen [4]), functional MRI (fMRI) studies have shown functional connections of the striatum with several core nodes of the so-called Default-Mode Network (DMN), including connections of the ventral striatum with the Precuneus/Posterior Cingulate Cortices (PC/PCC) and the anterior cingulate [3], [5], of the anterior caudate with the inferior parietal and Dorso-Medial Prefrontal Cortex (DMPFC) [6], [7], and of the putamen with the ventral anterior cingulate [8].

The DMN is a set of functionally interconnected brain structures, characterized by greater activation during rest relative to goal-directed task, which has gained a particular attention, due to its apparently central role in the coordination of sensori-motor and cognitive goal-directed activities, with intrinsic functions related to the self-awareness [9]–[11].

Highlighting the central role of the DMN, variations in this network have been already reported in both normal ageing [12] and in a wide range of neurological and psychiatric disorders, including Alzheimer’s disease, mild cognitive impairment, amyotrophic lateral sclerosis, schizophrenia, depression, epilepsia, and Parkinson’s disease [9].

In symptomatic HD patients, besides the alteration of the functional connections with the striatum directly induced by the degeneration of this structure, the previously shown atrophy of some DMN nodes (such as the anterior cingulate prefrontal and parietal regions) [13], [14], may hamper DMN function.

As the striatal-DMN loop has been found to play a role in complex cognitive functions in both physiological (such as in expert chess playing [15]) and pathological (such as depression [16] and drug addiction [17]) conditions, its alterations may have a role in determining and/or modulating cognitive disturbances in HD.

Consistent with this hypothesis, a recent study probing the DMN in pre-manifest HD (using independent component analysis applied to data from a fMRI activation study) found a reduced correlation of the posterior part of the DMN with the DMPFC and of the left inferior parietal cortex (lIPC, correlating with the reaction time at an attention task), and of the posterior cingulate with the anterior DMN subsystem [18], in the context of an increased connectivity between anterior and posterior DMN subsystems.

However, another study on a large sample of prodromal HD patients, focusing on correlation of Stroop-interference related functional connectivity with depressive symptoms, did not show differences in the DMN components [19], thus questioning the presence of these alterations.

To date, to the best of our knowledge, studies of the DMN integrity in symptomatic HD patients are lacking.

Aim of the present study was to evaluate in symptomatic HD patients the integrity of the DMN, as assessed by a functional connectivity analysis of Resting State fMRI (RS-fMRI) data, and to assess possible correlations between DMN alterations and motor or cognitive symptoms.

To discriminate apparent atrophy-related reductions of functional connectivity in DMN nodes from primary functional changes, DMN analysis was performed controlling for the effect of gray matter (GM) volume at voxel level [20], an approach which allows to disentangle functional and structural changes in the involved structures.

## Methods

### Ethics Statement

The present study was approved by the local Institutional Review Board of the University “Federico II” of Naples, and informed written consent was obtained from all participants.

### Subjects

Twenty-six symptomatic HD patients, included in the European Huntington’s Disease Network’s REGISTRY [21], and 22 normal volunteers (NV) of comparable age and gender were enrolled. All subjects were right-handed.

Patients scored positive on a molecular test with >38 CAG repeats in the huntingtin gene, and disease duration was retrospectively measured based on the age at which, according to the rater, the first motor, cognitive, or behavioral signs of HD appeared.

NV had no history of psychiatric or neurological disorders, of substance abuse, or treatment with medications active on the CNS.

Within one week from the MRI study an experienced neurologist examined the patients and recorded the motor part of the Unified HD Rating Scale (UHDRS), along with the Total Functioning Capacity [22]. The UHDRS cognitive subscore (an indicator of ‘executive functioning’, obtained summing the scores reported at the Stroop [23], phonological Verbal Fluency [24] and Symbol Digit Modality [25] tests) was also available in 16 patients.

The UHDRS motor subscore ranges from 0 to 124, with increasing scores reflecting more severely impaired motor function, while increasing UHDRS cognitive subscores reflect relatively preserved cognitive functions.

Additionally, the Beck Depression Inventory (BDI) Scale [26] and the Folstein’s Mini Mental State Examination (MMSE) [27] were available in 17 and 22 patients, respectively. BDI values below 10 are considered normal, while values between 11 and 17 indicate mild, 18–23 moderate, and above 23 severe depression.

MMSE has decreasing values with increasing dementia level, values above 26 being considered as normal.

### MRI Data Acquisition

All MRI studies were carried out at three Tesla on the same MRI scanner (Trio, Siemens Medical Systems, Erlangen, Germany).

Acquisition included a structural T1w volume for brain tissue volume assessment by segmentation, and a set of T2*-weighted volumes for RS-fMRI analysis, acquired within the same scanning session.

Structural T1w volume were acquired by three-dimensional Magnetization-Prepared RApid Gradient-Echo sequence (TE = 3.4 ms; TR = 1900 ms; TI = 900 ms; Flip Angle = 9°, FOV = 250; slice plane = axial; slice thickness = 1 mm; voxel size = .98×.98×1.00 mm^3^; number of slices = 160).

T2*-weighted volumes were acquired by echo-planar imaging (EPI) sequence (axial orientation, TR = 2500 ms, TE = 40 ms, FOV = 192 mm, 64×64 matrix, 30 slices, slice thickness 4 mm, gap 1 mm, 128 time points).

During the MRI study the subjects were laying supine with the head lightly fixed by straps and foam pads to minimize head movement, and were asked to relax with eyes closed.

### MRI Data Analysis

To obtain brain tissue maps needed for subsequent seed definition and voxel-based correction of RS-fMRI data for GM atrophy (see below), structural data were segmented and registered to the standard MNI space using the fast diffeomorphic registration algorithm (Diffeomorphic Anatomical Registration using Exponentiated Lie algebra - DARTEL) [28], implemented in the Statistical Parametric Mapping software package (SPM8 - http://www.fil.ion.ucl.ac.uk/spm Wellcome Trust Centre for Neuroimaging, University College London). For all the DARTEL preprocessing steps the default SPM8 parameters were used.

The details of the segmentation procedure are reported separately in the supporting information, along with the results of the assessment of GM atrophy, performed using Voxel-Based Morphometry (VBM, performed to verify the consistency of our segmentation results with previous studies of GM atrophy in HD, see Figures S1 and S2, and table S1 in File S1).

RS-fMRI data pre-processing was carried out using both Analysis of Functional NeuroImaging (AFNI, http://afni.nimh.nih.gov/afni/) and fMRIb Software Library (FSL, http://fsl.fmrib.ox.ac.uk/fsl/fslwiki/FSL).

After discarding the first 5 time points to allow for instability of the initial MRI signal, leaving 123 time points, the following pre-processing steps were applied: interpolation of fMRI time series to correct for acquisition time-shift, rigid-body volume registration of each fMRI volume to the first time point of the corresponding fMRI series (3dvolreg function in AFNI [29], a method for motion correction which proved stable and independent from image noise [30]), despiking (3dDespike function in AFNI: for each voxel, flattening of the outliers in the time series using a hyperbolic tangent function), spatial smoothing using a Gaussian kernel of FWHM 5 mm, highpass temporal filtering (Gaussian-weighted least-squares straight line fitting, with sigma = 50 s), and Gaussian lowpass temporal filtering (HWHM 2.8 s).

Registration of the EPI series to the standard MNI space was performed using as a proxy for each study the averaged motion-corrected and despiked time series, which were preliminarily spatially normalized [31] to the EPI template provided with SPM8, without introducing geometric distortions. Resulting normalized mean fMRI volumes were then averaged to generate a local, site- and study-specific, EPI template. Nonlinear components were not used for template creation in order to preserve group affine geometry.

Averaged fMRI time series were then normalized to the local EPI template including geometric distortions (16 parameters), and the deformation matrices were applied to each time point of the resulting motion-corrected and despiked EPI series.

For all normalization procedures, default SPM8 parameters were used.

Normalized volumes were then interpolated to a 3×3×3 mm^3^ voxel size, and accuracy of the spatial normalization was visually assessed by an experienced operator, blind to the condition of the subject.

For seed-based analysis, a region of interest sampling the posterior main hub of the DMN, which includes the posterior cingulate and the ventral portion of the precuneus, was obtained by eroding with a 6×6×6 mm^3^ kernel the map of Brodmann Areas 23 and 31, as defined in the MNI space in the WFU PickAtlas version 2.3 (http://fmri.wfubmc.edu/software/PickAtlas [32], [33]).

It should be noted that, given the lack of a clear boundary between the part of precuneus connected to the DMN and the remaining of BA7 [34], we chose to restrict our seed region to BAs 23 and 31, and for the purposes of the present work this ROI is defined PC/PCC, as BA31 is a transition zone from the medial parietal areas to the posterior cingulate cortex which has been suggested to belong to both posterior cingulate and precuneate cortices, [35], [36].

For each study the PC/PCC region of interest was then masked by the corresponding normalized GM map, to avoid contamination from CSF and WM signal, and the mean across the ROI voxels was derived from each time point to obtain the PC/PCC time-activity curve.

The map of correlation with the time-activity curve of the PC/PCC region was produced by a general linear model analysis, using FMRIB’s Improved Linear Model with local autocorrelation correction [37].

Accordingly, for each study, along with the seed time activity curve, nine sources of spurious variance were included in a multiple linear regression analysis, along with their temporal derivatives, which included the six head motion parameters (translation along and rotation around the three orthogonal axes), as estimated from the previous rigid-body registration procedure, and the whole-brain, CSF and WM signals (obtained averaging the corresponding voxels as derived from the segmented maps, after an erosion with a 6×6×6 mm^3^ kernel).

Movement parameters and global brain tissue signal curves were included to focus on region-specific signal changes, removing the effects of signal fluctuations due to respiratory and cardiac activity, or head movement, which involve the whole brain and are unrelated to cerebral activity [10].

Additionally, to minimize potentially confounding effects of different degrees of movement between the two groups, which have shown the potential to introduce artefactual correlations and reduce existing ones in functional connectivity studies [38], [39], a quality assurance and artifact rejection software (ART, www.nitrc.org/projects/artifact_detect/) was used to identify in each study time points with excessive (>2 mm) mean voxel displacement, as calculated for intracranial voxels derived from the translation and rotation parameters obtained from the coregistration procedure, and/or with abnormal changes in global mean signal intensity over the intracranial volume (>5std), suggestive of movement artifacts.

The list of volumes with excessive movement or abnormal signal intensity, as provided by ART, was then expanded including the volume preceding and the two volumes following each time points, to allow for temporal smoothing of BOLD data performed in the pre-processing steps, and reestablishment of steady-state spins. The resulting list of time points was finally used to remove from the subsequent first-level analysis the time points prone to potential motion-related artifacts [38].

The resulting Z (Gaussianised T/F) statistic maps of correlation with PC/PCC underwent between-groups (second-level) analysis, which was carried out using the Biological Parametric Mapping (BPM) toolbox Rel. 1.5d (http://www.ansir.wfubmc.edu) [20]. BPM allowed an analysis of covariance within SPM8, with age and sex as nuisance covariates, controlling for atrophy on a voxel-wise basis by using each subject’s normalized GM probability maps as a covariate.

To perform BPM, the normalized GM maps, modulated by their jacobian determinants, were interpolated to a 3×3×3 mm^3^ voxel size and smoothed using a 7-mm FWHM isotropic Gaussian kernel, to obtain a comparable degree of smoothing as the fMRI data (resulting Resolution Elements were 32.9 voxels for smoothed GM maps and 28.5 voxels for fMRI Z maps, respectively), divided by ICV to control for head size, and included as a voxel-based nuisance regressor in the analysis.

Between-group differences were probed using a threshold of p<.05, controlled for FWE rate by multiple comparison correction at cluster level (following pre-selection of voxels surviving an uncorrected threshold of p<.001). Both positive (NV>HD) and negative (NV<HD) contrasts were probed, and resulting statistically significant clusters were located anatomically according to [40], using the Anatomy toolbox [41] for SPM8.

Results are reported both with and without control for GM atrophy, to allow comparison with studies not incorporating the correction for GM volume differences.

As a substantial proportion of the patients was under pharmacological treatment with potential effects on the CNS (see results), an ancillary analysis was performed separately including only on the drug-free patients, to rule out the effect of drugs on the fMRI results.

### Correlation of DMN with Clinical Data

We explored the correlation between the clinical data and the Z values of the DMN regions which showed significant differences between NV and HD patients. Accordingly, for each cluster which was significantly different in HD patients compared to NV at the RS-fMRI analysis, the 1^st^ eigenvariate (a measure of the central tendency of the cluster, which is robust to the heterogeneity of the response within the cluster) of the z values was extracted,and Spearman’s non-parametric correlation coefficient was used to assess its correlation versus clinical data.

Tested clinical variables included number of CAG repeats, age, disease duration, severity of motor and cognitive symptoms as assessed by the motor and cognitive UHDRS subscores (Stroop, Verbal Fluency, and Symbol Digit Modality), and by the Total Functioning Capacity score, as well as depression as assessed by the BDI score, and overall cognitive status as assessed by the MMSE.

Results were considered significant for p<.05, corrected for multiple comparisons according to Bonferroni.

## Results

Clinical data of normal subjects and patients are summarized in Table 1.

**Table 1:** Subject demographics and clinical variables.

	Normal Volunteers (n = 22)		HD patients (n = 26)		
	Mean±SD	Range	Mean±SD	Range	P-value
**Age (years)**	39.1±13.7	18–63	43.7±11.6	18–68	0.22*
**Sex (M/F)**	11/11		16/10		0.42#
**CAG repeat size**	N.A.	–	46.5±5.3	41–65	
**Disease Duration (y)**	N.A.	–	4.4±3.1	0.5–13.0	
**Total Functioning Capacity**	N.A.	–	10.1±2.8	4–13	
**UHDRS motor score**	N.A.	–	20.0±10.9	4–53	
**MMSE (n = 22)**	N.A.	–	25.2±3.6	15–30	
**Stroop**	N.A.	–	117.4±40.9	40–191	
**Digit Symbol Modality**	N.A.	–	17.2±7.6	6–38	
**Verbal Fluency**	N.A.	–	14.0±6.7	1–28	
**BDI (n = 17)**	N.A.	–	13.1±10.8	0–34	

SD: standard deviation.

N.A.: not applicable.

* at Student’s t-test.

# at Chi-squared test.

Fourteen of the patients were drug naïve, three assumed associations of neuroleptics, benzodiazepines and selective serotonin reuptake inhibitors (SSRI), the remaining nine were in treatment with antidepressants (SSRI or noradrenaline and serotonin reuptake inhibitors) and/or low doses of typical neuroleptics for hyperkinesias.

Ten of the 22 tested patients had a MMSE below 26, and 5 of the 17 patients who underwent the BDI showed clinical signs of depression (BDI>16).

### RS-fMRI

Mean displacement of intracranial voxels, based on the translation and rotation parameters estimated from the rigid-body registration procedure using ART, was significantly different between HD patients and NV (0.33±0.26 vs. 0.14±0.09 mm, respectively, p = 0.001).

In the NV group the pattern of positive and negative (anti-) correlations with the PC/PCC seed (shown in Figure S3 of the supporting information, with corresponding clusters reported in Tables S2 and S3 in File S1) was consistent with previous studies of the DMN [42]. Main regions directly correlating with PCC included bilaterally inferior parietal regions (mainly encompassing the angular gyrus), the fusiform and middle temporal gyri, the anterior cingulate, and the medial temporal cortex (parahippocampal and amygdala), along with infratentorial correlations mainly with lobules 9, and crus I and II of the cerebellum. Anti-correlations were present bilaterally with clusters located in the inferior parietal area (encompassing mainly the supramarginal gyrus) and in the insula, with extension in the inferior perirolandic cortex, more pronounced on the right, which have been observed in previous studies [43], [44] to be part of the so-called Task-Positive Networks [10], [45], [46].

**Figure 1:**
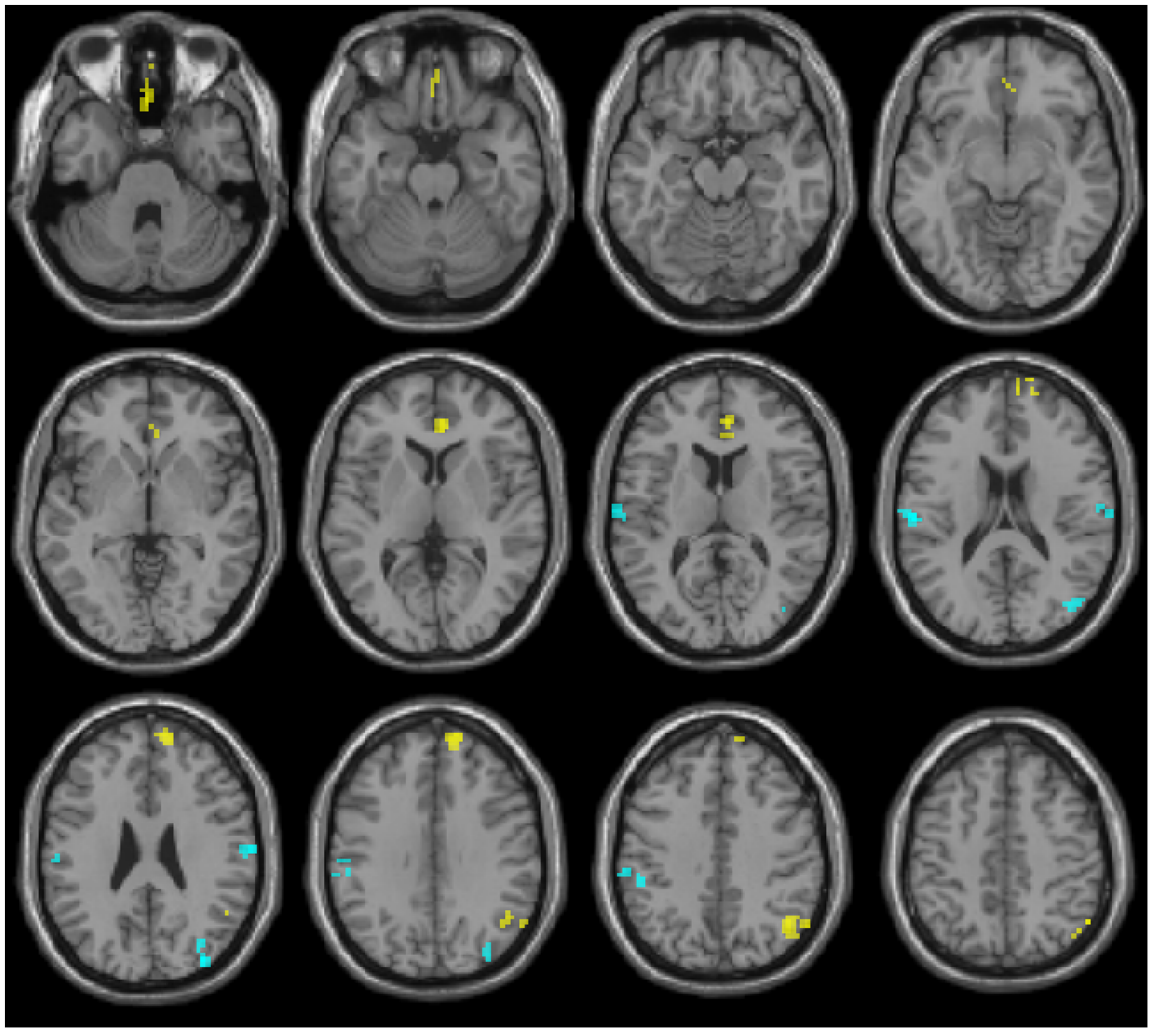
Results of resting-state fMRI analysis, corrected for atrophy. Clusters resulting from the NV>HD (yellow) and HD>NV (cyan) between-group contrasts, corrected for differences in GM volume. Results are superimposed for anatomical reference to a single subject T1-weighted volume in the standard Montreal Neurological Institute stereotactic space. Significance for all clusters is p<.05 FWE-corrected at cluster level. Patient’s right is at the observer’s right. Axial planes are sampled every 7 mm, starting at Z = −30 mm.

Clusters showing a significantly different correlation with PC/PCC in HD patients compared to NV, controlled for atrophy (both contrasts, NV>HD and HD>NV), are reported in tables 2 and 3, respectively, and shown in Figure 1 (results uncontrolled for GM atrophy are also reported within supporting information, in Figures S2 and S4).

**Table 2:** Results of the NV>HD between-group contrast.

	Cluster volume (mm^3^)				MNI		
	RS-fMRI	BPM		T	X	Y	Z
**VMPFC**	5157	2538	**Right Anterior Cingulate**	5.40	6	42	9
			**Right Mid Orbital Gyrus**	4.45	6	36	−12
			**Left Anterior Cingulate**	4.18	0	42	−6
			**Left Mid Orbital Gyrus**	3.52	0	51	−6
**rDMPFC**	2565	2025	**Right Superior Medial Gyrus**	5.90	15	60	30
			**Right Superior Frontal Gyrus**	5.05	18	69	18
**rANG**	2484	2025	**Right Angular Gyrus**	5.59	54	−63	45
			**Right Inferior Parietal Lobule**	4.87	57	−63	39
**STRIATUM**	2862	–	**Right Caudate Nucleus**	5.78	9	15	−3
			**Left Caudate Nucleus**	5.03	−12	18	−6

For each cluster the size before and after correction for atrophy using BPM is reported. For each involved structure, the maximum T-value and the corresponding coordinates (distances from the anterior commissure in mm) in the MNI space are reported.

**VMPFC**: Ventral Medial PreFrontal Cortex; **rDMPFC**: right Dorso-Medial PreFrontal Cortex; **rANG**: right Angular Gyrus.

Structures involved by each cluster are reported with the corresponding MNI X/Y/Z coordinates and maximum T value.

Anatomical labeling is according to [40].

**Table 3:** Results of the HD>NV between-group contrast.

	Cluster volume (mm^3^)				MNI		
	RS-fMRI	BPM		T	X	Y	Z
**lSMG**	4023	2403	**Left Postcentral Gyrus**	5.79	−57	−18	21
			**Left SupraMarginal Gyrus**	5.52	−60	−27	42
			**Left Inferior Parietal Lobule**	4.70	−51	−33	39
**rOCC**	1701	1674	**Right Middle Occipital Gyrus**	5.62	36	−84	27
**lOCC**	2052	–	**Left Superior Occipital Gyrus**	5.17	−15	−102	12
			**Left Middle Occipital Gyrus**	4.45	−45	−72	6
			**Left Inferior Occipital Gyrus**	3.87	−45	−75	−6
**lINS**	1296	–	**Left Insula**	4.54	−42	−3	−3
**rSMG**	1971	1134	**Right SupraMarginal Gyrus**	4.78	66	−15	27
			**Right Postcentral Gyrus**	4.48	69	−15	21
**SMA**	1134	–	**Left SMA**	4.75	0	−3	63
			**Right SMA**	3.77	6	6	57

For each cluster the size before and after correction for atrophy using BPM is reported. For each involved structure, the maximum T-value and the corresponding coordinates (distances from the anterior commissure in mm) in the MNI space are reported.

**lSMG** and **rSMG**: left and right SupraMarginal Gyrus, respectively; **rOCC** and **lOCC**: right and left Occipital cortex, respectively; **lINS**: left Insula; **SMA**: Supplementary Motor Area.

Structures involved by each cluster are reported with the corresponding maximum T value.

Anatomical labeling is according to [40].

HD showed, compared to NV, a decreased correlation with the PC/PCC in three clusters, respectively at the level of the ventral medial prefrontal cortex (VMPFC, including anterior cingulate and subgenual cortex), the DMPFC on the right (rDMPFC), and the right inferior parietal cortex, mainly involving the angular gyrus (rANG).

The HD>NV contrast resulted in three clusters. Two of these, involving bilaterally the inferior parietal cortices at the level of the supramarginal gyri (rSMG and lSMG), were due to decreased anti-correlation (in regions showing negative correlation in NV), while in the occipital cortex there was an expansion of the inferior parietal component of the DMN, only significant on the right when GM volume was taken into account using BPM. Additionally, two smaller clusters of reduced anti-correlation in the left Insula and in the supplementary motor area were not significant when atrophy was accounted for.

The ancillary analysis performed on the fMRI data of the drug-free patients provided substantially overlapping results (Figure S5 of the supporting information).

### Correlation between Imaging and Clinical Data

The VMPFC cluster showed a significant positive correlation (p = .005) with the cognitive subset of the UHDRS, demonstrating reduced connectivity in more compromised patients. Post-hoc testing with sub-items of the UHDRS-cognitive score disclosed this significance to be mainly due to a correlation with the total Stroop score (p = .0002).

No other correlation was present between fMRI data and other indexes of motor impairment, cognitive impairment, and depression.

## Discussion

This is, to the best of our knowledge, the first fMRI study of DMN in symptomatic HD patients.

In symptomatic HD patients morphometric studies have demonstrated, besides the obvious striatal damage, a diffuse pattern of atrophy involving cortical structures [14], [47], [48], more prominent in the sensori-motor cortices, but extending to virtually all cortical regions with disease progression [48], [49], and already present in preclinical stages of the disease [50].

The presence of atrophy in regions overlapping those where RS-fMRI alterations are found, raises the question of the origin of the observed fMRI changes. The reduced functional connectivity in the DMN nodes may in fact be due in principle to reduced signal variations related to tissue loss, which may hamper our ability to assess the connectivity of these structures.

BPM analysis allowed us to disentangle these two contributions, confirming that the reduced correlation to PC/PCC in several nodes of the DMN (VMPFC, rSMPFC, and right inferior parietal cortex), the reduced anti-correlations in the supramarginal gyri, and the cluster of increased correlation in the right occipital cortex, were at least partially unrelated to the atrophy of these structures, confirming the functional nature of the involvement of these DMN and task-positive network nodes.

On the other hand, BPM analysis unveiled that the connectivity loss in caudate nuclei, along with the loss of negative correlation in SMA left insula and left occipital cortex, which was present when the analysis was performed without control for GM volume, can be explained by the atrophy of these structures.

Beside primary neuronal dysfunction in the DMN nodes, at least two different mechanisms should be considered as potentially generating the reduced direct or inverse correlation. These include the cumulative effects of more diffuse cortical structural alterations, and the loss of a possible striatal regulatory role (due to primary striatal degeneration).

The first mechanism is in line with the observation that diffuse disconnection mechanisms seem to play a major role in cognitive impairment in HD [51].

The latter proposed mechanism, on the other hand, is in line with the known regulatory role of dopaminergic innervation on the DMN. Pharmacological dopaminergic challenges [52]–[55] and positron emission tomography (PET) studies [56] have in fact linked the dopaminergic system to DMN deactivation during cognitive task performance. In particular, l-DOPA challenge has demonstrated a modulatory effect on the DMN [52], [54], reducing functional connectivity between PC/PCC and dorsal caudate, and between PC/PCC and other DMN nodes, including ACC/medial PFC and ventral striatum, while dopamine depletion has shown a reduced deactivation in the DMN during frontal tasks [57], and l-DOPA challenge restored normal task-induced deactivation in posterior midline and lateral parts of DMN in parkinsonian patients [58]. In line with these findings, apomorphine (a D1 and D2 agonist) challenge during frontal task has shown an effect only on the anterior portion of the DMN, including the ventromedial prefrontal cortex (VMPFC) [53], a region which has also shown increased task-related deactivations facilitating sensorimotor processing speed when modafinil (a norepinephrine/dopamine transporter inhibitor) is administered [59].

Additionally, several evidences support the role of the genetic functional variations of dopamine-inactivating enzymes [60], [61] and of D2 receptors [62] in modulating the DMN.

In addition, it should also be considered that alterations of the cerebral vasculature reactivity may also play a role in altering our ability to exploit the blood-oxygenation-level-dependent fMRI signal changes to detect neural activity variations. These alterations are indeed present in HD [63], [64], possibly related to altered nitric oxide regulation [65], linked to inflammatory phenomena and glial activation that take place in associative regions already in the preclinical phases of the disease [66]. It should be however considered that the direct relationship between DMN alterations and cognitive performance that we found in these patients mitigates against the hypothesis that DMN down-regulation may be only an artefactual consequence of changes in vascular reactivity, unrelated to functional connectivity changes in the disease.

The changes in connectivity within the VMPFC showed indeed a significant correlation with rather specific cognitive functions in HD. The lack of correlation between the DMN alterations and the MMSE, as opposed to the more “frontal-driven” UHDRS cognitive subset, highlights in fact a selective role of these changes in the alterations of frontal executive cognitive processes.

Functional MRI connectivity studies, focusing on connectivity changes during the execution of frontal tasks, have been performed in preclinical [67] and symptomatic [68] HD patients.

In presymptomatic mutation carriers it has been shown, during high-load working memory tasks, a lack of the increase in coherence in frontostriatal and frontoparietal networks which is normally associated to working memory tasks in NV [67], while in symptomatic HD patients, impaired functional connectivity between anterior cingulate and lateral prefrontal regions has been demonstrated when performing relevant frontal tasks [68].

Taken together, these findings suggest the presence of a dysregulation of cortico-striatal networks in HD, which may play a role in the cognitive impairment of these patients, in line with diffusion tensor studies, which have highlighted in HD a damage not limited to the subdivisions of the striatum subserving motor tasks, but also of those linked to cognitive functions [69], [70].

DMN modifications, although with heterogeneous patterns, have been demonstrated in several neurodegenerative and non-primarily neurodegenerative disorders where cognitive alterations are present [9], raising the question of the specificity of the present DMN alterations, rather than an association of these alterations with cognitive deficits “per se”.

However, the existing evidences of a direct connection of the basal ganglia with the main DMN nodes, and, more importantly, of a functional interaction between hubs of the motor pathways and the DMN (specifically, in HD patients deep brain stimulation of the external globus pallidus modulated the metabolic pattern within the DMN [71]), suggest that these alterations can be disease-specific, and possibly connected to dysfunction of the complex mechanisms connecting motor networks and cognitive functions in HD, although further studies are needed to assess the specificity to HD of the present findings.

Consistent with this view, lower functional connectivity in motor regions [72] during a low-demand alertness task, and hyperactivation of medial pre- and supplementary motor areas during a time-discriminant task [73], have been detected in the preclinical stages of HD.

The correlation of the VMPFC dysfunction with the cognitive performance, as tested by the cognitive subset of the UHDRS, is mainly driven by the correlation with the results at the Stroop test, a test which can be influenced by an appropriate functioning of the DMN [74]. Our findings expand the results of previous studies which have shown in HD a correlation of the performance at the Stroop test with the degree of atrophy in specific thalamic nuclei [75], including the dorso-medial nuclei, which project to the prefrontal cortices, and suggest a more complex alteration of the basal ganglia-thalamocortical circuitry.

Indeed these findings are in keep with previous studies of the functional connectivity correlates of the Stroop interference-related activations, which have also shown in presymptomatic carriers of the HD mutation a synchronism in the VMPFC node of the DMN [19] with Stroop task, correlating with depressive symptoms.

More in general, the assessment of DMN integrity in presymptomatic patients has provided so far somewhat conflicting results.

In particular, contrary to the findings of the previously cited study by Wolf et Al. [18], which reported evidences of altered connectivity between the anterior and posterior DMN subsystems, a functional connectivity study, performed on a larger sample of presymptomatic HD patients [19], has found no significant differences in the synchronism or in the spatial extent of the two DMN subsystems between presymptomatic patients and NV.

Several methodological differences, coupled to the different sample sizes, may at least partly explain the differences between the results of these two studies (and in turn may limit the comparability of the results of these two works with ours), including the experimental conditions (stroop vs. alertness), the inclusion in the second level analysis of different behavioural/cognitive variables as covariates of no interest, and insufficient artifact detection and motion correction measures.

However, it should be noted that both works provide hits of an alteration of the relationship between posterior and anterior nodes of the DMN, as in one case, the different relationships existing between depressive symptoms and Stroop interference-related activity in the two DMN subsystems may indicate already a disturbance of their normal connection, while in the other, the reduced representations of the posterior cingulate in the anterior subsystem, and of the anterior medial prefrontal cortex in the posterior subsystem are in line with our findings in symptomatic patients.

Although frontal structures have been quite constantly shown functional alterations in previous activation studies of HD, care should be taken in correlating results obtained in different phases of the disease. Previous functional studies have consistently shown cingulate gyrus dysfunction, ranging from hypoactivation of the anterior cingulate in some phases of preclinical HD [76], correlating with years to onset [73], [77], to more frequent hyperactivation findings in more advanced phases of the disease [63], [78], [79].

This raises the question of a possible heterogeneity of the patterns of connectivity changes (including those involving the DMN) expanding into the clinical phase of the disease, which can hardly be assessed when analyzing cross sectional data, as acknowledged also in previous fMRI studies [73], [77], [80]. Accordingly, longitudinal studies should help assess possible modifications of these alterations of the DMN throughout the clinical phases of the disease.

The PC/PCC seems to have a specific anti-correlation with the prefrontal-based motor control circuits, apparently negatively predicting their activity (as defined by Granger causality analyses [44]. The clusters of reduced anticorrelation in the supramarginal/postcentral regions may represent reduced activity of the DMN in counter-acting the sensorimotor network (which show a substantial hyperactivation at functional connectivity studies already in the preclinical phases of the disease in both executive and cognitive motor nodes, depending on the task demands on motor control [81], [82]), either to allow for compensatory changes or for a failure of the DMN itself.

Although the first hypothesis could be favored by the fact that we did not observe a correlation between these anticorrelations with the PC/PCC and the degree of motor impairment, it should be kept in mind, when interpreting the anticorrelations, that their overall strength is weaker than the direct correlations within the DMN, thus limiting our ability to detect it, and to detect their correlation to motor tasks. Also, a possible insufficient specificity of the UHDRS motor subset, a rating scale essentially targeted to clinical use, should be considered when interpreting these results.

Small clusters of increased connectivity with PC/PCC were present in HD patients in the occipital cortex, adjacent to main posterior nodes of DMN (figure 1) in areas which in the NV group were neither significantly correlated nor anti-correlated with the PC/PCC.

It is tempting to speculate that these changes may represent direct compensatory increases in connectivity within the posterior part of the DMN, to compensate for deficits of the anterior nodes, similar to what has been hypothesized in healthy ageing [83] and in widespread brain tissue damage, like in traumatic brain injury [84]. However, further studies are needed to clarify the mechanisms underlying these increases in connectivity and their significance.

HD individuals are more likely to suffer from sleepiness [85]. As sleepiness during resting state scan can slightly modify the synchrony of brain networks [86], this may represent a possible source of differences between the two groups, as the patients may have more easily slept during the scan.

Although we did not collect a sleepiness scale following the scan, we verified that the subjects were awake immediately before the start and immediately after the end of the resting-state sequence (by interacting via the scanner interphone). Given the short duration of the resting-state scan (less than 5′), we do not expect any of the subjects to have slept during the scan. Furthermore, following each study we verified if the subject had the impression to have slept at any time during the scan.

Our patient population included two patients with juvenile HD (jHD) (age at onset of 11 and 19, with 65 and 50 CAG repeats, respectively), a form which may be clinically different from the adult-onset one [87], and may have more frequent psychiatric and cognitive disturbances already at onset [88].

To rule out an effect of this variant on our results, we performed an ancillary analysis (both of VBM and RS-fMRI data) excluding these two cases. The results substantially overlapped those obtained on the whole HD group in all the analyses performed (data not shown), with the exceptions of the cluster of reduced connectivity in the basal ganglia, which was split in two clusters which both approached significance (p = .06 on the right and.07 on the left).

Although these results demonstrate that our findings are not significantly influenced by the inclusion of this subgroup, the small number of jHD patients and the age differences precluded to perform a direct comparison between adult HD and jHD patients, so that further work is needed to assess possible differences between these two subgroups.

Finally, as a substantial proportion (14/26) of our patients was being pharmacologically treated for motor and/or psychiatric symptoms, a potential interference of the therapeutic regimen on the DMN synchronism could be expected.

However, although the heterogeneity of the treatments in our group precludes an analysis of specific drug effects, it should be noted that drug effects can hardly explain our results, as the results of the ancillary analysis performed only on the drug-free patient subgroup (Figure S5 of the supporting information) presented a substantial spatial overlap with those obtained on the whole sample. The minor differences which can be appreciated, compared to present results, when analyzing only the drug-free patients (limited to an increased cranial extension of the rDMPFC and a reduced extension of the lOCC clusters), are likely due to the different degrees of freedom (28 vs. 42) in the of the two analyses. However, future studies should be carried out in larger drug-naïve patient populations to fully rule out any potential effect of drugs on these results.

### Conclusions

DMN dysfunction is present in symptomatic HD patients, expanding beyond regions of alteration found in the preclinical stages of the disease [72], and correlates to cognitive (and not motor) disturbances. This dysfunction is not directly related to the atrophy of the involved cortical nodes, suggesting either a mechanism related to a possible role of the striatum in regulating a subset of the DMN in the normal brain, or the effect of a more widespread neuronal damage, which is known to occur in the clinical phases of the disease.

Further studies are needed to clarify the mechanisms underlying these alterations, including an assessment of structural disconnection (e.g. by diffusion tensor imaging) to evaluate its role in the loss of synchrony within the DMN, and longitudinal studies which will help assess possible modifications of the pattern of these alterations throughout the disease course, as well as their role in the clinical history of the disease.

## Supporting Information

Figure S1 **Clusters of gray matter loss in HD patients.** Results are displayed for p = 0.05 FWE-corrected at cluster level, projected onto the surface of a standard brain in the Montreal Neurological Institute sterotactic space. No region showed a significantly increased GM volume in HD patients compared to normal volunteers.
(TIF)

Figure S2 **GM loss and DMN alterations in HD.** Clusters of significant GM loss in HD (red), along with clusters of significant differences, uncorrected for atrophy, in correlation with PC/PCC between HD patients and NV (yellow for NV>HD and cyan for HD>NV, respectively). Results are superimposed for anatomical reference to a single subject T1-weighted volume in the standard Montreal Neurological Institute sterotactic space. Significance is p<0.05 FWE-corrected at cluster level. Patient’s right is at the observer’s right. Axial planes are sampled every 7 mm, starting at Z = −30. It can be appreciated a substantial overlap of the VBM and RS-fMRI findings in the caudate nuclei.
(TIF)

Figure S3 **Correlations with PC/PCC in the normal volunteers.** Areas showing a significant positive (overlaid in red) or negative (overlaid in blue) correlation with the PC/PCC seeds in Normal Volunteers. Results are displayed for p = 0.05 FWE-corrected at cluster level, projected onto the surface of a standard brain in the Montreal Neurological Institute sterotactic space.
(TIF)

Figure S4 **Results of resting-state fMRI analysis, uncorrected for atrophy.** Differences in correlation with PC/PCC between HD patients and NV. Voxels showing significant positive (red) and negative (blue) correlation with PC/PCC in Normal Volunteers, along with clusters resulting from the NV>HD (yellow) and HD>NV (cyan) between-group contrasts, uncorrected for atrophy. Results are superimposed for anatomical reference to a single subject T1-weighted volume in the standard Montreal Neurological Institute sterotactic space. Significance for all clusters is p<0.05 FWE-corrected at cluster level. Patient’s right is at the observer’s right. Axial planes are sampled every 7 mm, starting at Z = −30 mm.
(TIF)

Figure S5 **Resting-state fMRI ancillary analysis in untreated patients.** “Glass brain” representation of the results of the ancillary analysis performed on the subset of 12 patients who were not being pharmacologically treated at the time of the scan. The clusters of significant differences in correlation with PC/PCC between HD patients and NV (upper row NV>HD, lower row HD>NV), corrected for GM volume, are reported for the drug-free patients (left column) and for the whole set of patients (right column) for comparison. Significance is p<0.05 FWE-corrected at cluster level for all images (following pre-selection of voxels surviving an uncorrected threshold of p<0.005 for drug-free patients, and of p<0.001 for whole set, to allow for reduced sample size). For the axial and coronal projections, patient’s right is at the observer’s right.
(TIF)

Text S1 **Description of VBM analysis.**
(DOC)
