# Supplementary figures and images for: Default-Mode Network Changes in Huntington’s Disease: An Integrated MRI Study of Functional Connectivity and Morphometry
Source: PLoS One. 2013 Aug 19;8(8):e72159. doi: 10.1371/journal.pone.0072159 (PMC3747049; doi:10.1371/journal.pone.0072159)

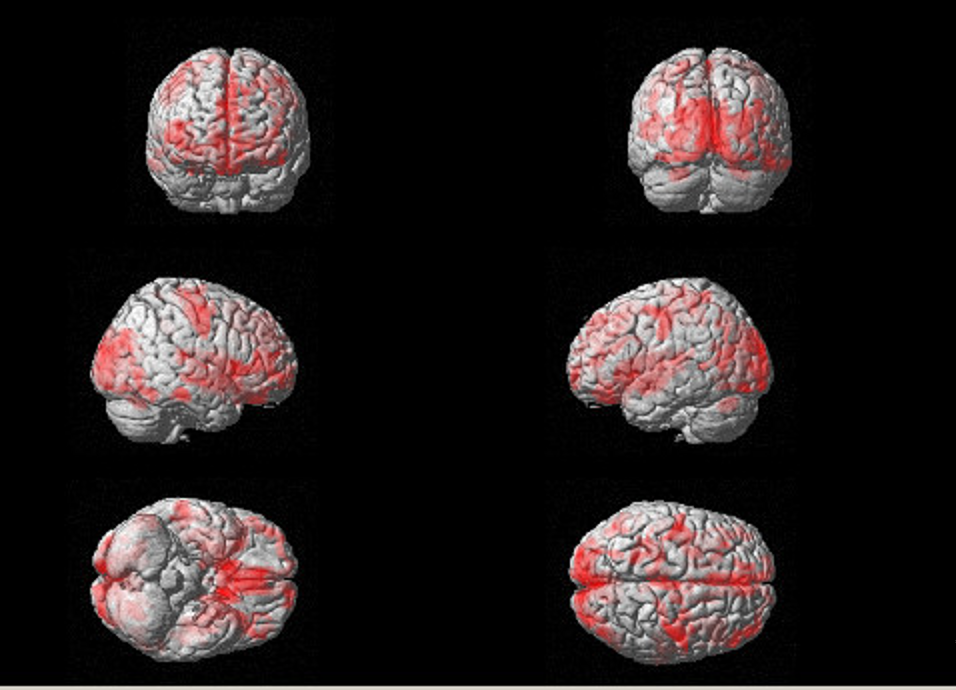

Supplement: Figure S1 — Clusters of gray matter loss in HD patients. Results are displayed for p = 0.05 FWE-corrected at cluster level, projected onto the surface of a standard brain in the Montreal Neurological Institute sterotactic space. No region showed a significantly increased GM volume in HD patients compared to normal volunteers. (TIF) [file pone.0072159.s001.tif]

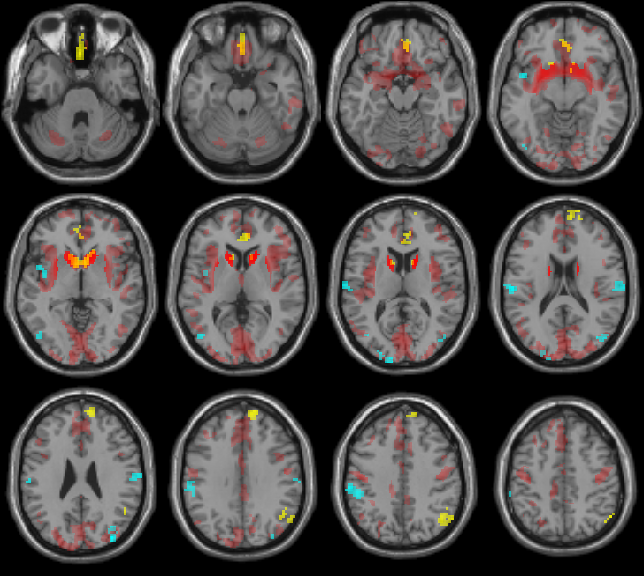

Supplement: Figure S2 — GM loss and DMN alterations in HD. Clusters of significant GM loss in HD (red), along with clusters of significant differences, uncorrected for atrophy, in correlation with PC/PCC between HD patients and NV (yellow for NV>HD and cyan for HD>NV, respectively). Results are superimposed for anatomical reference to a single subject T1-weighted volume in the standard Montreal Neurological Institute sterotactic space. Significance is p<0.05 FWE-corrected at cluster level. Patient’s right is at the observer’s right. Axial planes are sampled every 7 mm, starting at Z = −30. It can be appreciated a substantial overlap of the VBM and RS-fMRI findings in the caudate nuclei. (TIF) [file pone.0072159.s002.tif]

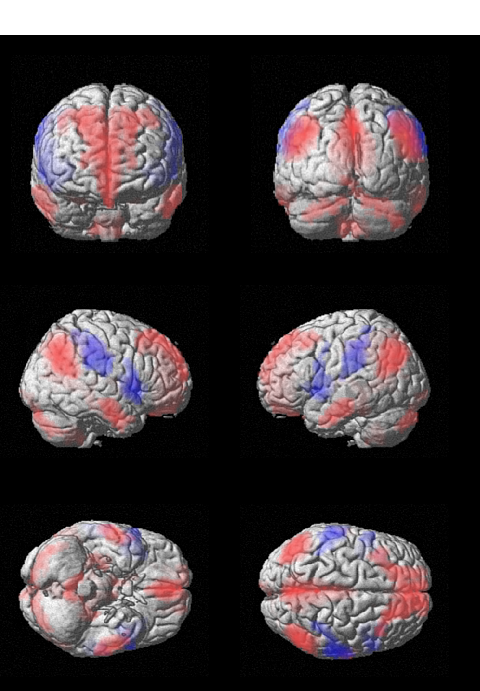

Supplement: Figure S3 — Correlations with PC/PCC in the normal volunteers. Areas showing a significant positive (overlaid in red) or negative (overlaid in blue) correlation with the PC/PCC seeds in Normal Volunteers. Results are displayed for p = 0.05 FWE-corrected at cluster level, projected onto the surface of a standard brain in the Montreal Neurological Institute sterotactic space. (TIF) [file pone.0072159.s003.tif]

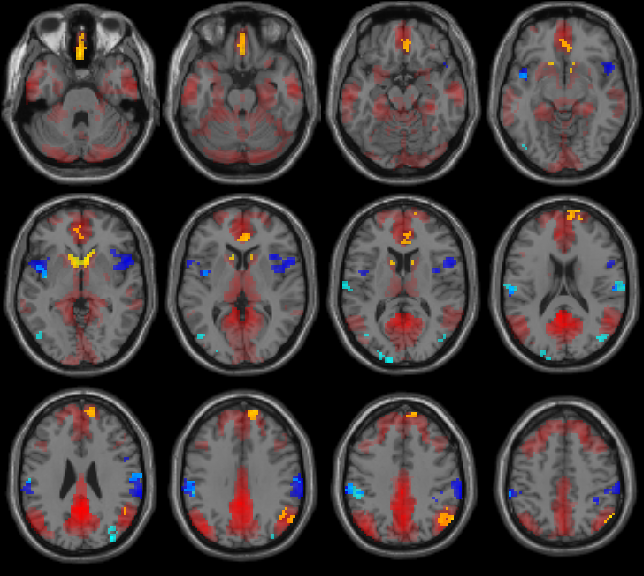

Supplement: Figure S4 — Results of resting-state fMRI analysis, uncorrected for atrophy. Differences in correlation with PC/PCC between HD patients and NV. Voxels showing significant positive (red) and negative (blue) correlation with PC/PCC in Normal Volunteers, along with clusters resulting from the NV>HD (yellow) and HD>NV (cyan) between-group contrasts, uncorrected for atrophy. Results are superimposed for anatomical reference to a single subject T1-weighted volume in the standard Montreal Neurological Institute sterotactic space. Significance for all clusters is p<0.05 FWE-corrected at cluster level. Patient’s right is at the observer’s right. Axial planes are sampled every 7 mm, starting at Z = −30 mm. (TIF) [file pone.0072159.s004.tif]

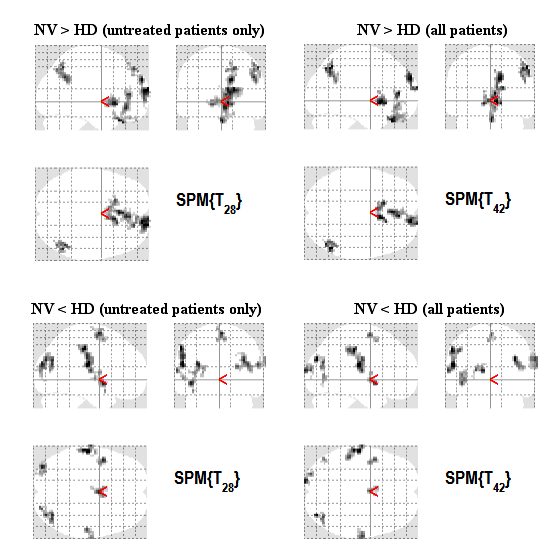

Supplement: Figure S5 — Resting-state fMRI ancillary analysis in untreated patients. “Glass brain” representation of the results of the ancillary analysis performed on the subset of 12 patients who were not being pharmacologically treated at the time of the scan. The clusters of significant differences in correlation with PC/PCC between HD patients and NV (upper row NV>HD, lower row HD>NV), corrected for GM volume, are reported for the drug-free patients (left column) and for the whole set of patients (right column) for comparison. Significance is p<0.05 FWE-corrected at cluster level for all images (following pre-selection of voxels surviving an uncorrected threshold of p<0.005 for drug-free patients, and of p<0.001 for whole set, to allow for reduced sample size). For the axial and coronal projections, patient’s right is at the observer’s right. (TIF) [file pone.0072159.s005.tif]
