# Supplementary material for: Default-Mode Network Changes in Huntington’s Disease: An Integrated MRI Study of Functional Connectivity and Morphometry
Source: PLoS One. 2013 Aug 19;8(8):e72159. doi: 10.1371/journal.pone.0072159 (PMC3747049; doi:10.1371/journal.pone.0072159)
Supplement: Text S1 — Description of VBM analysis. (DOC) [file pone.0072159.s006.doc]

**Default-mode network changes in Huntington’s Disease: an integrated MRI study of functional connectivity and morphometry.**

***^1^Mario Quarantelli, ^2^Elena Salvatore, ^3^Sara Maria Delle Acque Giorgio, ^2^Alessandro Filla, ^3^Amedeo Cervo, ^2^Cinzia Valeria Russo, ^3^Sirio Cocozza, ^2^Marco Massarelli, ^3^Arturo Brunetti, ^2^Giuseppe De Michele***

^1^Biostructure and Bioimaging Institute, National Research Council, Naples, Italy

^2^Department of Neurosciences, Reproductive Sciences and Odontostomatology, University “Federico II”, Naples, Italy

^3^Department of Advanced Biomedical Sciences, University “Federico II”, Naples, Italy

**SUPPORTING INFORMATION S1:**

**VBM**

Structural data were analyzed using a fast diffeomorphic registration algorithm (Diffeomorphic Anatomical Registration using Exponentiated Lie algebra - DARTEL) and VBM , implemented in the Statistical Parametric Mapping software package (SPM8 - <http://www.fil.ion.ucl.ac.uk/spm> Wellcome Trust Centre for Neuroimaging, University College London). For all the DARTEL preprocessing steps the default SPM8 parameters were used.

DARTEL proved a superior registration accuracy compared to conventional approaches to spatial normalization of T1w-volumes VBM, resulting in more sensitive VBM analysis .

Briefly, the DARTEL procedure is based on a preliminary segmentation into GM and white matter (WM) of the T1w-volumes using the unified segmentation model as implemented in SPM8 , GM and WM segments are then simultaneously coregistered and the resulting flow fields are applied to the rigidly-aligned segments to warp them to the common DARTEL space. A local GM template is then generated through an iterative nonlinear registration , which is then normalized to the standard anatomical space of the Montreal Neurological Institute , and the resulting deformations are applied to the GM volume of each subject.

The normalized GM maps (1.5x1.5x1.5mm^3^ voxel size) are then visually assessed, to ensure good quality of the normalization, and manually edited where needed to remove possible residual islands of extra-cerebral tissues incorrectly classified as GM.

Resulting maps are then modulated by the Jacobian determinants derived from the spatial normalization procedure, to preserve the local GM volumes, and then smoothed using a 6‑mm FWHM isotropic Gaussian kernel to reduce confounding by individual variations in gyral anatomy and to render the data more normally distributed as per the Gaussian random field model underlying the statistical process used for adjusting p-values.

For each study, total intracranial volume was then calculated, on the non-normalized segmented volumes, as the number of the voxels where the sum of GM, WM and CSF probabilities exceeded 50%.

Normalized modulated GM maps were statistically analyzed using the general linear model based on the random Gaussian field theory . The design matrix was constructed to test for regional differences in GM between the HD group and normal subjects. To take into account their effect on brain tissue volumes, age and sex were entered as nuisance regressors (confounding covariates) in an analysis of covariance, while total intracranial volume was entered in the model to normalize for head size. Regression analysis was carried out on voxels surviving a threshold of 20% of GM probability.

**VBM Results**

VBM results are shown in Figure S1. Corresponding regions of atrophy are reported in Table S1.

The pattern of atrophy in HD patients is in agreement with previous VBM studies of HD . In particular, our results demonstrate a widespread, mainly supratentorial, subcortical and cortical atrophy, with the main clusters of significant GM loss involving bilaterally, beside the striatum, the perirolandic and medial occipital cortices, as well as the insulae.

Testing for the inverse contrast (HD > NV) did not result in any significant cluster of increased GM density in HD patients.

**Table S1**

***Results of the VBM analysis.***

|  |  |  | **MNI** | | |
| --- | --- | --- | --- | --- | --- |
| **Cluster volume (mm^3^)** |  | **T** | **X** | **Y** | **Z** |
| 110477 | Right Caudate Nucleus | 17.99 | 12 | 17 | 9 |
|  | Left Caudate Nucleus | 16.56 | -11 | 17 | 0 |
|  | Left Putamen | 13.79 | -24 | 0 | -5 |
|  | Right Putamen | 13.28 | 20 | 9 | -8 |
| 67797 | Left Calcarine Gyrus | 7.79 | 0 | -76 | 10 |
|  | Left Postcentral Gyrus | 7.47 | -47 | -15 | 40 |
|  | Left Superior Occipital Gyrus | 7.37 | -18 | -94 | 19 |
|  | Left Cuneus | 6.71 | 0 | -81 | 16 |
|  | Left Lingual Gyrus | 6.70 | 0 | -79 | -2 |
|  | Left Middle Occipital Gyrus | 6.63 | -24 | -84 | 24 |
| 8056 | RightPrecentral Gyrus | 7.42 | 42 | -16 | 51 |
|  | Right Postcentral Gyrus | 6.45 | 48 | -10 | 34 |
| 4374 | Left Middle Temporal Gyrus | 4.92 | -44 | -64 | 6 |
|  | Left Angular Gyrus | 4.43 | -48 | -66 | 33 |
| 2268 | Left Cerebellum | 4.33 | -26 | -69 | -27 |
| 2035 | Left Inferior Frontal Gyrus | 4.57 | -45 | 26 | 24 |
|  | Left Middle Frontal Gyrus | 4.25 | -38 | 45 | 6 |
| 1860 | Right Superior Temporal Gyrus | 4.14 | 54 | -19 | 0 |
|  | Right Middle Temporal Gyrus | 4.05 | 56 | -25 | -6 |
| 1860 | Right Inferior Temporal Gyrus | 5.03 | 60 | -25 | -21 |
| 1738 | Right Middle Cingulate Cortex | 3.97 | 6 | -34 | 33 |
|  | Right Precuneus | 3.65 | 6 | -43 | 43 |
|  | Left Posterior Cingulate Cortex | 3.63 | 2 | -39 | 31 |

Clusters showing significantly decreased gray matter volume in HD patients (p<0.05, FWE- corrected at cluster level) are reported, along with the involved GM volume (in mm^3^), and the corresponding maximum T value.

No significant differences emerged when probing the opposite (HD > NV) contrast.

Anatomical labeling is according to .

**Table S2**

***Positive correlations with PC/PCC in normal volunteers***

| **Cluster volume (mm^3^)** |  |  | **MNI** | | |
| --- | --- | --- | --- | --- | --- |
|  |  | **T** | **X** | **Y** | **Z** |
| 79380 | Left Posterior Cingulate Cortex | 25.09 | 0 | -51 | 30 |
|  | Left Precuneus | 24.92 | -6 | -57 | 24 |
|  | Left Middle Cingulate Cortex | 21.96 | 0 | -33 | 39 |
|  | Left Cuneus | 19.82 | -6 | -69 | 24 |
|  | Right Cuneus | 19.27 | 9 | -63 | 21 |
|  | Right Precuneus | 15.94 | 6 | -54 | 9 |
|  | Left Lingual Gyrus | 15.69 | -9 | -51 | 3 |
|  | Right Lingual Gyrus | 12.40 | 9 | -42 | 3 |
|  | Right Middle Cingulate Cortex | 12.05 | 0 | -21 | 30 |
| 66231 | Left Anterior Cingulate Cortex | 13.76 | -6 | 42 | 18 |
|  | Left Mid Orbital Gyrus | 13.40 | 0 | 51 | -9 |
|  | Right Anterior Cingulate Cortex | 12.89 | 6 | 42 | 9 |
|  | Right Superior Medial Gyrus | 12.49 | 3 | 51 | 3 |
| 16686 | Right Angular Gyrus | 16.07 | 48 | -66 | 39 |
|  | Right Middle Temporal Gyrus | 13.38 | 51 | -54 | 18 |
| 16173 | Left Middle Occipital Gyrus | 17.65 | -42 | -72 | 33 |
|  | Left Angular Gyrus | 13.61 | -51 | -66 | 24 |
| 7857 | Left Inferior Temporal Gyrus | 13.01 | -57 | -6 | -30 |
|  | Left Middle Temporal Gyrus | 10.45 | -60 | -12 | -24 |
| 7695 | Right Middle Temporal Gyrus | 11.73 | 60 | -9 | -24 |
|  | Right Inferior Temporal Gyrus | 7.42 | 54 | 0 | -39 |
|  | Right Medial Temporal Pole | 6.92 | 54 | 12 | -33 |
| 6831 | Right Cerebellum, Lobule VIIa | 9.45 | 27 | -87 | -30 |
| 5103 | Left Cerebellum, Lobule IX | 10.30 | -6 | -57 | -48 |
|  | Right Cerebellum, Lobule IX | 9.83 | 12 | -48 | -48 |
| 3969 | Left Cerebellum, Lobule VIIa | 8.44 | -30 | -81 | -33 |

Clusters showing significant positive correlation with PC/PCC in NV (p<0.05, FWE corrected at voxel level) are reported, along with the involved GM volume (in mm^3^). For each involved structure, the maximum T-value and the corresponding coordinates (distances from the anterior commissure in mm) in the MNI space are reported.

Anatomical labeling is according to .

**Table S3**

***Negative correlations with PC/PCC in normal volunteers***

| **Cluster volume (mm^3^)** |  | | |  | **MNI** | | |  | |
| --- | --- | --- | --- | --- | --- | --- | --- | --- | --- |
|  |  | **T** | | | **X** | **Y** | **Z** | | |
| 8505 | Right Postcentral Gyrus | | 6.36 | | 66 | -15 | 30 | |  |
|  | Right Inferior Parietal Lobule | | 4.90 | | 39 | -42 | 48 | |  |
|  | Right SupraMarginal Gyrus | | 4.32 | | 45 | -33 | 42 | |  |
| 8019 | Right Rolandic Operculum | | 6.02 | | 54 | 12 | 0 | |  |
|  | Right Inferior Frontal Gyrus (p. Opercularis) | | 5.87 | | 54 | 12 | 9 | |  |
|  | Right Insula Lobe | | 5.20 | | 48 | 3 | 3 | |  |
| 4374 | Left SupraMarginal Gyrus | | 6.46 | | -60 | -27 | 36 | |  |
|  | Left Inferior Parietal Lobule | | 5.00 | | -57 | -33 | 45 | |  |
|  | Left Postcentral Gyrus | | 4.80 | | -60 | -21 | 21 | |  |
| 3861 | Left Insula Lobe | | 6.73 | | -45 | 6 | -6 | |  |
|  | Left Inferior Frontal Gyrus (p. Opercularis) | | 4.43 | | -57 | 12 | 3 | |  |

Clusters of significant negative correlation with PC/PCC (p<0.05, FWE corrected at cluster level), along with the involved GM volume (in mm^3^). For each involved structure, the maximum T-value and the corresponding coordinates (distances from the anterior commissure in mm) in the MNI space are reported.

Anatomical labeling is according to .

**REFERENCES**

**1. Ashburner J (2007) A fast diffeomorphic image registration algorithm. Neuroimage 38: 95-113.**

**2. Ashburner J, Friston KJ (2000) Voxel-based morphometry--the methods. Neuroimage 11: 805-821.**

**3. Ashburner J, Friston KJ (2005) Unified segmentation. Neuroimage 26: 839-851.**

**4. Good CD, Johnsrude IS, Ashburner J, Henson RN, Friston KJ, et al. (2001) A voxel-based morphometric study of ageing in 465 normal adult human brains. Neuroimage 14: 21-36.**

**5. Klein A, Andersson J, Ardekani BA, Ashburner J, Avants B, et al. (2009) Evaluation of 14 nonlinear deformation algorithms applied to human brain MRI registration. Neuroimage 46: 786-802.**

**6. Mazziotta J, Toga A, Evans A, Fox P, Lancaster J, et al. (2001) A probabilistic atlas and reference system for the human brain: International Consortium for Brain Mapping (ICBM). Philos Trans R Soc Lond B Biol Sci 356: 1293-1322.**

**7. Friston KJ, Holmes AP, Poline JB, Grasby PJ, Williams SC, et al. (1995) Analysis of fMRI time-series revisited. Neuroimage 2: 45-53.**

**8. Rosas HD, Liu AK, Hersch S, Glessner M, Ferrante RJ, et al. (2002) Regional and progressive thinning of the cortical ribbon in Huntington's disease. Neurology 58: 695-701.**

**9. Douaud G, Gaura V, Ribeiro MJ, Lethimonnier F, Maroy R, et al. (2006) Distribution of grey matter atrophy in Huntington's disease patients: a combined ROI-based and voxel-based morphometric study. Neuroimage 32: 1562-1575.**

**10. Rosas HD, Salat DH, Lee SY, Zaleta AK, Pappu V, et al. (2008) Cerebral cortex and the clinical expression of Huntington's disease: complexity and heterogeneity. Brain 131: 1057-1068.**

**11. Ille R, Schafer A, Scharmuller W, Enzinger C, Schoggl H, et al. (2011) Emotion recognition and experience in Huntington disease: a voxel-based morphometry study. J Psychiatry Neurosci 36: 383-390.**

**12. Tzourio-Mazoyer N, Landeau B, Papathanassiou D, Crivello F, Etard O, et al. (2002) Automated anatomical labeling of activations in SPM using a macroscopic anatomical parcellation of the MNI MRI single-subject brain. Neuroimage 15: 273-289.**
